# Delayed Ventricular Tachycardia After Prophylactic Doses of Droperidol in Patients With Mild QT Interval Prolongation Due to Preoperative Medication

**DOI:** 10.7759/cureus.15560

**Published:** 2021-06-10

**Authors:** Kenichi Takechi, Ichiro Shimizu

**Affiliations:** 1 Anesthesia, Matsuyama Red Cross Hospital, Ehime, JPN

**Keywords:** droperidol, ventricular tachycardia, prolonged qt interval, ponv prophylaxis, aprindine

## Abstract

Prophylactic doses of droperidol are effective in preventing postoperative nausea and vomiting (PONV). However, due to concerns of QT interval prolongation and ventricular arrhythmias, the safety of droperidol for PONV prophylaxis has been debated.

A 70-year-old woman was scheduled for total knee arthroplasty. She had a history of aortic valve replacement. Oral aprindine (40 mg/day) was prescribed. Preoperative electrocardiogram showed mild QT interval prolongation (QTc = 475 ms). Anesthesia was induced using propofol, remifentanil, and rocuronium, and maintained using desflurane, remifentanil, and a bolus dose of rocuronium.

The surgery was uneventful. At the time of skin closure, droperidol (1.25 mg) was administered intravenously for PONV prophylaxis. Twenty-three minutes after administration of droperidol, a sudden onset of premature cardiac contraction was observed, which progressed directly to ventricular tachycardia and atrioventricular block. Arrhythmia due to droperidol-induced QT interval prolongation was strongly suspected. Intravenous magnesium sulfate (2 g) and atropine (0.5 mg) were administered immediately. The ventricular tachycardia resolved quickly after the magnesium injection. Following the resolution of the arrhythmia, the patient was extubated.

The patient experienced ventricular tachycardia after a prophylactic dose of droperidol that resulted from QT interval prolongation due to the preoperative medication. It may be prudent to avoid even low-dose droperidol in the background of already present QT prolongation, especially when multiple putative QT-prolonging drugs are used.

## Introduction

Postoperative nausea and vomiting (PONV) is a distressing and common complication in patients undergoing surgery with general anesthesia; thus, PONV prophylaxis is considered an essential part of high-quality perioperative care. Prophylactic droperidol (0.625-1.25 mg) is effective for the prevention of PONV, with a number needed to treat of approximately five [1]. However, in 2001, a “black box” specific warning was added to the droperidol package insert due to concerns of prolonged QT interval and ventricular arrhythmias, specifically torsades de pointes. Since then, the safety of small doses (less than 2.5 mg) of droperidol for PONV prophylaxis has been debated [2,3].

## Case presentation

**Figure 1:**
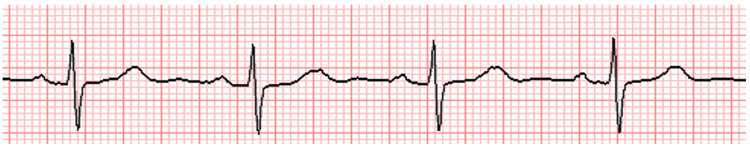
Preoperative electrocardiography. Mild QT interval prolongation (QTc = 475 ms, correction with Bazett’s formula).

A 70-year-old woman (height 151 cm, weight 55 kg) with American Society of Anesthesiologists (ASA) class 3 was scheduled for total knee arthroplasty due to the diagnosis of knee osteoarthritis. The patient had a history of aortic valve stenosis and aortic valve replacement. Postoperatively, she experienced supraventricular tachycardia that was controlled with oral aprindine (40 mg/day). No new arrhythmias were reported after the introduction of aprindine (Class 1b antiarrhythmic agent). Preoperative laboratory findings were within normal limits, except the electrocardiogram which showed mild QT interval prolongation (QTc = 475 ms, correction with Bazett’s formula) (Figure 1).

A standard anesthetic protocol was implemented, including noninvasive arterial blood pressure monitoring, electrocardiography (ECG), and oxygen saturation (SpO_2_) measurement upon arrival at the operating room. Anesthesia was induced using propofol (2 mg/kg), remifentanil (0.3 μg/kg/min), and rocuronium (0.8 mg/kg), and was maintained using desflurane (4%-6%), remifentanil (0.15-0.3 μg/kg/min), and bolus dose of rocuronium (10 mg). After induction of anesthesia, dexamethasone (3.3 mg) was administered for PONV prophylaxis. The following ventilator settings were used: tidal volume 7 mL/kg predicted body weight, inspired O_2_ fraction 0.6 with air, and inspiratory fresh gas flow 2 L/min. The respiratory rate was adjusted to 8-16 breaths/minute to maintain an end-tidal carbon dioxide (ETCO_2_) pressure of 30-45 mmHg. After anesthetic induction and securing the airway with the ProSeal laryngeal mask airway (LMA ProSeal, Teleflex, Ireland), femoral nerve and popliteal sciatic nerve blocks (0.75% ropivacaine 10 mL each nerve) were performed under ultrasound guidance.

**Figure 2:**
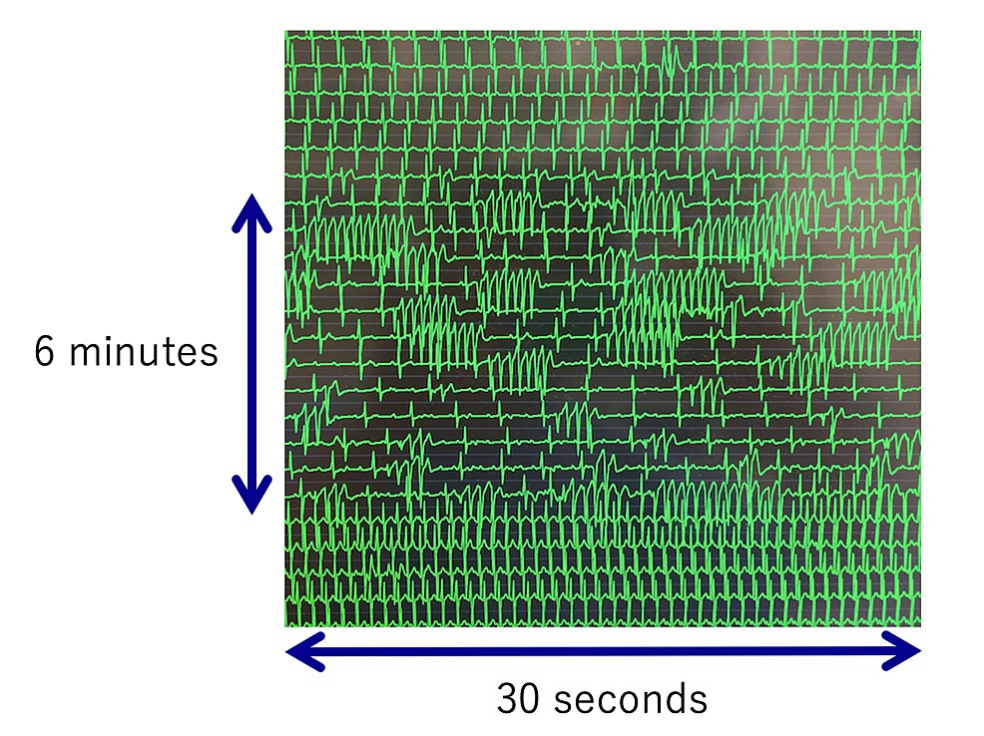
Electrocardiography of the arrhythmias. Sudden onset of premature cardiac contraction was observed, which progressed directly to polymorphic ventricular tachycardia and atrioventricular block. The arrhythmia lasted approximately six minutes and resolved quickly after the injection of magnesium.

The surgery was uneventful and lasted 1 hour and 49 minutes. Intraoperative bleeding volume was 290 mL. At the time of skin closure, droperidol (1.25 mg) was administered intravenously for PONV prophylaxis. Twenty-three minutes after the administration of droperidol, the patient was waiting for postoperative knee radiography and was still under general anesthesia. At that time, the systolic blood pressure was approximately 100 mmHg and the heart rate was approximately 50 beats/minute. Sudden onset of premature contraction was observed, which progressed directly to ventricular tachycardia and atrioventricular block (Figure 2).

Arrhythmia due to droperidol-induced QT interval prolongation was strongly suspected. Intravenous magnesium sulfate (2 g) and atropine (0.5 mg) were administered immediately. The ventricular tachycardia resolved quickly after the magnesium injection. Results of the arterial blood gas analysis were unremarkable. Following the resolution of the arrhythmia, the patient was extubated. The patient underwent 24-hour postoperative ECG and SpO_2_ monitoring. No new arrhythmias were observed. The patient was discharged after rehabilitation with a good postoperative course.

## Discussion

In the present case, ventricular tachycardia appeared at 23 minutes after prophylactic administration of droperidol. In the simplified algorithm for PONV prophylaxis, three types of prophylaxis are recommended for women [4]. In a recent review of over 20,000 patients receiving over 35,000 doses of droperidol, no patient developed polymorphic ventricular tachycardia, although over 500 patients reported QT interval prolongation [5]. Thus, for our patient, although we were aware of the presence of mild QT interval prolongation, we went ahead with droperidol for PONV prophylaxis.

In this case, due to a previous history of cardiac surgery, desflurane was used for the cardioprotective effect of volatile anesthetic [6]. All volatile anesthetics, especially isoflurane and desflurane, have been found to prolong the QT interval [7]. To prevent PONV and arrhythmia, propofol should be used for the maintenance of general anesthesia. In addition, recent guidelines for PONV recommend a multimodal approach, including 5-hydroxytryptamine receptor antagonists and neurokinin 1 receptor antagonists [8].

Long QT syndrome is a condition in which repolarization of the heart after a heartbeat is affected. This results in an increased risk of an irregular heartbeat, which can result in sudden death. There are several subtypes of long QT syndrome. These can be broadly split into those caused by genetic mutations and those caused by other factors, such as taking a QT interval-prolonging drug [9]. In the present patient, QT interval prolongation was observed after the initiation of oral aprindine, suggesting drug-induced long QT syndrome. Aprindine is a Class 1b antiarrhythmic agent, which is known to shorten the action potential. QT interval prolongation and polymorphous ventricular tachycardia have been reported as side effects of aprindine [10]. It is possible that preoperative oral aprindine and intraoperative desflurane may have affected the proarrhythmic effects of droperidol. The safety of the combination of perioperative medication and droperidol for PONV prophylaxis requires further investigation.

There were no further arrhythmic events in this case. The serum levels of droperidol are the highest immediately after administration. However, some reports suggest that the QT interval prolongation effect of droperidol may persist for several hours after administration [11]. Based on our case, we suggest that patients who receive droperidol should be monitored for several hours postoperatively.

## Conclusions

The patient experienced delayed ventricular tachycardia after a prophylactic dose of droperidol that resulted in QT interval prolongation due to preoperative aprindine. The safety profile of perioperative medication and the prophylactic dose of droperidol requires further study. It may be prudent to avoid even low-dose droperidol in the background of already present QT prolongation, especially when multiple putative QT-prolonging drugs are used.
